# Supplementary material for: HIVprotI: an integrated web based platform for prediction and design of HIV proteins inhibitors
Source: J Cheminform. 2018 Mar 9;10:12. doi: 10.1186/s13321-018-0266-y (PMC5845081; doi:10.1186/s13321-018-0266-y)
Supplement: Supplementary file 2 — Additional file 2. Source code of HIVProtI web server. [file 13321_2018_266_MOESM2_ESM.zip › HIVprotI_Source-code/hivprotI/weka-3-6-6/doc/weka/filters/unsupervised/attribute/ClusterMembership.html]

ClusterMembership


---


|  |  |  |
| --- | --- | --- |
| **Overview** \| **Package** \| **Class** \| **Tree** \| **Deprecated** \| **Index** \| **Help** \| **Weka's home** | |  |
| **PREV CLASS**   **NEXT CLASS** | **FRAMES**    **NO FRAMES**     **All Classes** |  |
| SUMMARY: NESTED \| FIELD \| CONSTR \| METHOD | DETAIL: FIELD \| CONSTR \| METHOD |  |


---


## weka.filters.unsupervised.attribute Class ClusterMembership

```
java.lang.Object
  weka.filters.Filter
      weka.filters.unsupervised.attribute.ClusterMembership
```

**All Implemented Interfaces:**: java.io.Serializable, CapabilitiesHandler, OptionHandler, RevisionHandler, UnsupervisedFilter

---

``` public class ClusterMembership extends Filter implements UnsupervisedFilter, OptionHandler ```

A filter that uses a density-based clusterer to generate cluster membership values; filtered instances are composed of these values plus the class attribute (if set in the input data). If a (nominal) class attribute is set, the clusterer is run separately for each class. The class attribute (if set) and any user-specified attributes are ignored during the clustering operation


Valid options are:

```
 -W <clusterer name>
  Full name of clusterer to use. eg:
   weka.clusterers.EM
  Additional options after the '--'.
  (default: weka.clusterers.EM)
```

```
 -I <att1,att2-att4,...>
  The range of attributes the clusterer should ignore.
  (the class attribute is automatically ignored)
```

Options after the -- are passed on to the clusterer.

**Version:**
:   $Revision: 1.16 $

**Author:**
:   Mark Hall (mhall@cs.waikato.ac.nz), Eibe Frank

**See Also:**: Serialized Form

---

**Constructor Summary**

`ClusterMembership()`


| **Method Summary** | |
| --- | --- |
| `boolean` | `batchFinished()`             Signify that this batch of input to the filter is finished. |
| `java.lang.String` | `densityBasedClustererTipText()`             Returns a description of this option suitable for display as a tip text in the gui. |
| `Capabilities` | `getCapabilities()`             Returns the Capabilities of this filter. |
| `Capabilities` | `getCapabilities(Instances data)`             Returns the Capabilities of this filter, makes sure that the class is never set (for the clusterer). |
| `DensityBasedClusterer` | `getDensityBasedClusterer()`             Get the clusterer used by this filter |
| `java.lang.String` | `getIgnoredAttributeIndices()`             Gets ranges of attributes to be ignored. |
| `java.lang.String[]` | `getOptions()`             Gets the current settings of the filter. |
| `java.lang.String` | `getRevision()`             Returns the revision string. |
| `java.lang.String` | `globalInfo()`             Returns a string describing this filter |
| `java.lang.String` | `ignoredAttributeIndicesTipText()`             Returns the tip text for this property |
| `boolean` | `input(Instance instance)`             Input an instance for filtering. |
| `java.util.Enumeration` | `listOptions()`             Returns an enumeration describing the available options. |
| `static void` | `main(java.lang.String[] argv)`             Main method for testing this class. |
| `void` | `setDensityBasedClusterer(DensityBasedClusterer newClusterer)`             Set the clusterer for use in filtering |
| `void` | `setIgnoredAttributeIndices(java.lang.String rangeList)`             Sets the ranges of attributes to be ignored. |
| `boolean` | `setInputFormat(Instances instanceInfo)`             Sets the format of the input instances. |
| `void` | `setOptions(java.lang.String[] options)`             Parses a given list of options. |

**Methods inherited from class weka.filters.Filter**

`batchFilterFile, filterFile, getOutputFormat, isFirstBatchDone, isNewBatch, isOutputFormatDefined, makeCopies, makeCopy, numPendingOutput, output, outputPeek, toString, useFilter, wekaStaticWrapper`

**Methods inherited from class java.lang.Object**

`equals, getClass, hashCode, notify, notifyAll, wait, wait, wait`

**Constructor Detail**

### ClusterMembership

```
public ClusterMembership()
```


**Method Detail**

### getCapabilities

```
public Capabilities getCapabilities()
```

:   Returns the Capabilities of this filter.

    :   **Specified by:**: `getCapabilities` in interface `CapabilitiesHandler` **Overrides:**: `getCapabilities` in class `Filter`
    :   **Returns:**: the capabilities of this object **See Also:**: `Capabilities`

---


### getCapabilities

```
public Capabilities getCapabilities(Instances data)
```

:   Returns the Capabilities of this filter, makes sure that the class is
    never set (for the clusterer).

    :   **Overrides:**: `getCapabilities` in class `Filter`
    :   **Parameters:**: `data` - the data to use for customization **Returns:**: the capabilities of this object, based on the data **See Also:**: `getCapabilities()`

---


### setInputFormat

```
public boolean setInputFormat(Instances instanceInfo)
                       throws java.lang.Exception
```

:   Sets the format of the input instances.

    :   **Overrides:**: `setInputFormat` in class `Filter`
    :   **Parameters:**: `instanceInfo` - an Instances object containing the input instance structure (any instances contained in the object are ignored - only the structure is required). **Returns:**: true if the outputFormat may be collected immediately **Throws:**: `java.lang.Exception` - if the inputFormat can't be set successfully

---


### batchFinished

```
public boolean batchFinished()
                      throws java.lang.Exception
```

:   Signify that this batch of input to the filter is finished.

    :   **Overrides:**: `batchFinished` in class `Filter`
    :   **Returns:**: true if there are instances pending output **Throws:**: `java.lang.IllegalStateException` - if no input structure has been defined: `java.lang.NullPointerException` - if no input structure has been defined,: `java.lang.Exception` - if there was a problem finishing the batch.

---


### input

```
public boolean input(Instance instance)
              throws java.lang.Exception
```

:   Input an instance for filtering. Ordinarily the instance is processed
    and made available for output immediately. Some filters require all
    instances be read before producing output.

    :   **Overrides:**: `input` in class `Filter`
    :   **Parameters:**: `instance` - the input instance **Returns:**: true if the filtered instance may now be collected with output(). **Throws:**: `java.lang.IllegalStateException` - if no input format has been defined.: `java.lang.NullPointerException` - if the input format has not been defined.: `java.lang.Exception` - if the input instance was not of the correct format or if there was a problem with the filtering.

---


### listOptions

```
public java.util.Enumeration listOptions()
```

:   Returns an enumeration describing the available options.

    :   **Specified by:**: `listOptions` in interface `OptionHandler`
    :   **Returns:**: an enumeration of all the available options.

---


### setOptions

```
public void setOptions(java.lang.String[] options)
                throws java.lang.Exception
```

:   Parses a given list of options.


    Valid options are:

    ```
     -W <clusterer name>
      Full name of clusterer to use. eg:
       weka.clusterers.EM
      Additional options after the '--'.
      (default: weka.clusterers.EM)
    ```

    ```
     -I <att1,att2-att4,...>
      The range of attributes the clusterer should ignore.
      (the class attribute is automatically ignored)
    ```

    Options after the -- are passed on to the clusterer.

    :   **Specified by:**: `setOptions` in interface `OptionHandler`
    :   **Parameters:**: `options` - the list of options as an array of strings **Throws:**: `java.lang.Exception` - if an option is not supported

---


### getOptions

```
public java.lang.String[] getOptions()
```

:   Gets the current settings of the filter.

    :   **Specified by:**: `getOptions` in interface `OptionHandler`
    :   **Returns:**: an array of strings suitable for passing to setOptions

---


### globalInfo

```
public java.lang.String globalInfo()
```

:   Returns a string describing this filter

    :   **Returns:**: a description of the filter suitable for displaying in the explorer/experimenter gui

---


### densityBasedClustererTipText

```
public java.lang.String densityBasedClustererTipText()
```

:   Returns a description of this option suitable for display
    as a tip text in the gui.

    :   **Returns:**: description of this option

---


### setDensityBasedClusterer

```
public void setDensityBasedClusterer(DensityBasedClusterer newClusterer)
```

:   Set the clusterer for use in filtering

    :   **Parameters:**: `newClusterer` - the clusterer to use

---


### getDensityBasedClusterer

```
public DensityBasedClusterer getDensityBasedClusterer()
```

:   Get the clusterer used by this filter

    :   **Returns:**: the clusterer used

---


### ignoredAttributeIndicesTipText

```
public java.lang.String ignoredAttributeIndicesTipText()
```

:   Returns the tip text for this property

    :   **Returns:**: tip text for this property suitable for displaying in the explorer/experimenter gui

---


### getIgnoredAttributeIndices

```
public java.lang.String getIgnoredAttributeIndices()
```

:   Gets ranges of attributes to be ignored.

    :   **Returns:**: a string containing a comma-separated list of ranges

---


### setIgnoredAttributeIndices

```
public void setIgnoredAttributeIndices(java.lang.String rangeList)
```

:   Sets the ranges of attributes to be ignored. If provided string
    is null, no attributes will be ignored.

    :   **Parameters:**: `rangeList` - a string representing the list of attributes. eg: first-3,5,6-last **Throws:**: `java.lang.IllegalArgumentException` - if an invalid range list is supplied

---


### getRevision

```
public java.lang.String getRevision()
```

:   Returns the revision string.

    :   **Specified by:**: `getRevision` in interface `RevisionHandler` **Overrides:**: `getRevision` in class `Filter`
    :   **Returns:**: the revision

---


### main

```
public static void main(java.lang.String[] argv)
```

:   Main method for testing this class.

    :   **Parameters:**: `argv` - should contain arguments to the filter: use -h for help


---


|  |  |  |
| --- | --- | --- |
| **Overview** \| **Package** \| **Class** \| **Tree** \| **Deprecated** \| **Index** \| **Help** \| **Weka's home** | |  |
| **PREV CLASS**   **NEXT CLASS** | **FRAMES**    **NO FRAMES**     **All Classes** |  |
| SUMMARY: NESTED \| FIELD \| CONSTR \| METHOD | DETAIL: FIELD \| CONSTR \| METHOD |  |


---
